# Accuracy and Variability of a Commercial Markerless Motion Capture System Compared to a Pressure Mat for Weight Distribution in Standing: Cross-Sectional Observational Study

**DOI:** 10.2196/73575

**Published:** 2025-12-02

**Authors:** Lisa Sheehy, Emma Gal-Dev, Heidi Sveistrup, Martin Bilodeau, Hillel Finestone

**Affiliations:** 1 Bruyère Health Research Institute Ottawa, ON Canada; 2 Interdisciplinary School of Health Sciences Faculty of Health Sciences University of Ottawa Ottawa, ON Canada; 3 School of Rehabilitation Sciences Faculty of Health Sciences University of Ottawa Ottawa, ON Canada; 4 Department of Systems and Computer Engineering Faculty of Engineering and Design Carleton University Ottawa, ON Canada; 5 School of Human Kinetics Faculty of Health Sciences University of Ottawa Ottawa, ON Canada; 6 Department of Medicine, Division of Physical Medicine and Rehabilitation Faculty of Medicine University of Ottawa Ottawa, ON Canada; 7 Physical Medicine and Rehabilitation Bruyère Health Ottawa, ON Canada

**Keywords:** balance, motion capture, rehabilitation, stroke, stroke rehabilitation, validation study, virtual reality, weight shift

## Abstract

**Background:**

Commercial markerless motion capture (MMC) systems show promise for use in rehabilitation and have been validated for the assessment of various parameters. However, no prior studies have evaluated MMC systems to detect stance asymmetry.

**Objective:**

The objective of this study was to assess the accuracy and variability of the Jintronix Weight Shift Tool MMC system to estimate the percentage of weight borne on each foot.

**Methods:**

Twelve healthy younger adults, 12 healthy older adults, and 12 people living with stroke were recruited for this cross-sectional study. The percentage of weight borne on each foot was simultaneously recorded by the Weight Shift Tool and a validated pressure mat during 2 series (raising the arm to capture the recording and without arm raise) with left lean, right lean, and equal stance. Agreement between the Weight Shift Tool and the pressure mat was assessed using Bland-Altman analyses.

**Results:**

Bias was greatest for older adults for all stances except for right lean without arm raise. Variability was greatest for people living with stroke for all stances except for left lean with arm raise. On average, the limits of agreement were narrower during equal stance. Although bias between the Weight Shift Tool and the pressure mat was small to moderate (0.0%-11.7%), the limits of agreement were wide (12.8%-33.6% above and below the bias).

**Conclusions:**

The Weight Shift Tool is not clinically acceptable for the estimation of the percentage of weight on each foot due to high variability. Investigation of other MMC systems is required to confirm the validity of MMC for clinical assessment.

## Introduction

The use of markerless motion capture (MMC) for tracking human movement has provided numerous possibilities for exergaming (exercise performed by playing video games that require physical activity), in that it allows for unrestricted movement unhindered by the need to wear a special garment, apply reflective dots, or hold a controller. With a video or infrared camera, software is used to detect the location of the main segments and joints of the body and follow their movements. Furthermore, software can track and record joint angles and changes in tilt from the vertical. These features may make the use of MMC technology helpful for the clinical assessment of range of motion and posture, as it provides an easier, faster, and cheaper means of assessment in comparison to marker-based motion capture systems [1,2]. In addition, MMC shows promising potential for application in stroke rehabilitation. Exercises performed with MMC technology may provide effective physiotherapy supplementation for rehabilitation after stroke, in a clinical setting or at home [3-7]. The use of MMC technology for telerehabilitation allows feedback to be provided during exercise and for clinicians to track progress from a distance, reducing the need for frequent in-person visits.

There is current interest in the development of accurate and reliable MMC technologies for home rehabilitation and physiotherapy supplementation. Recently, novel MMC systems using commercial infrared camera systems based on deep learning have been developed [8-10]. The most commonly used system in studies evaluating MMC for stroke rehabilitation is the Microsoft Kinect sensor (Microsoft Corp) [4]. The Microsoft Kinect v2 sensor has been tested in healthy individuals and people living with stroke. From testing with healthy individuals, it was deemed reliable for the assessment of gait (*r*=0.92 for gait velocity and *r*=0.81 for step length), and valid to assess velocity (no difference between Kinect and Vicon, *P*=.26) and acceleration (no difference between Kinect and Vicon, *P*=.42) of one’s center of gravity in the anteroposterior direction while performing a sit-to-stand task [11,12]. However, Yeung et al [13] found that the Kinect sensor was more accurate in measuring body sway in the mediolateral direction (mean difference of 2.0 mm between Kinect and Vicon) than in the anteroposterior direction (mean difference of 3.7 mm between Kinect and Vicon). Mobini et al [14] evaluated the Kinect sensor’s ability to monitor the progress of upper-body rehabilitation in people living with stroke. The Kinect sensor was able to reliably detect changes in mean hand velocity (*R*^2^=0.96), the logarithm of the median of the hand’s dimensionless jerk (*R*^2^=0.94), and the hand’s path curvature (*R*^2^=0.97) after 1 month of rehabilitation [14]. The Kinect v2 sensor also tested favorably against manual and digital goniometry for measuring shoulder joint angles in healthy individuals, with differences between readings generally less than 3 degrees except when measuring external rotation [15]. Overall, studies seem to agree that MMC, and specifically the Kinect sensor, shows promise for application as a clinical assessment and rehabilitation tool.

One commercial application of the Kinect v2 sensor for rehabilitation is the Jintronix Rehabilitation System (Jintronix Inc). In addition to using user location and body segment movement for exergaming, clinicians are provided with a measure of stance symmetry through the Weight Shift Tool. Although the use of MMC has been evaluated for many assessments, no studies have been found on its accuracy and variability in measuring weight distribution on each foot. Stance asymmetry is a clinically important measure in physiotherapy. Weight distribution on each foot is an aspect of balance function, which is necessary for independence in activities of daily living and fall prevention [16-18]. In fact, asymmetrical weight-bearing is a common result of stroke [19]. Furthermore, weight-bearing asymmetry can act as an indicator of loss of balance, is positively correlated with postural sway, and may be a useful outcome measure for individuals with lateropulsion [16,20,21]. Therefore, rehabilitation interventions often target improvements in balance and symmetry, both in static and dynamic conditions, as important components of the recovery of patients with stroke, orthopaedic injuries, and gait abnormalities [22-24]. Older adults may also experience declines in balance function as a result of physiological changes from aging [25]. Current assessment methods for weight-bearing asymmetry include the use of two digital bathroom scales, used by standing with one foot on each scale, which has been deemed accurate for patients with stroke (ranging from *F*_38_=0.006; *P*=.94 to *F*_38_=0.074; *P*=.79), although significant variability was noted between trials on the paretic (*F*_38_=3.47; *P*=.004) and nonparetic (*F*_38_=4.51; *P*=.002) sides [26]. An easy, accurate, and reliable measurement of weight distribution in standing could be used to assess and treat stance asymmetries, consequently improving the quality of life in people living with stroke. The validation of commercially available MMC technology, such as the Jintronix Weight Shift Tool, as an acceptable tool for the assessment of weight distribution in standing could be beneficial for clinicians and patient outcomes. Therefore, the purpose of this study was to investigate the ability of the Weight Shift Tool, which incorporates the Kinect v2 sensor, to assess weight distribution in standing by estimating the percentage of weight on each foot.

The objectives of this study were as follows:

1. To assess the accuracy and variability of the Weight Shift Tool, compared to a pressure mat;
2. To determine if raising one’s arm (to capture a recording) affected the accuracy or variability of the Weight Shift Tool;
3. To determine if there was a difference in the accuracy or variability of the Weight Shift Tool depending on participant group (younger adults, older adults, people living with stroke).

The hypotheses were as follows:

1. That the accuracy of the Weight Shift Tool is equivalent to a pressure mat for measuring weight distribution in standing and that the variability of the Weight Shift Tool (measured using the Bland-Altman limits of agreement) is less than 5%.
2. That raising one’s arm would demonstrate no significant effect on the accuracy or variability of the Weight Shift Tool.
3. That there would be no significant difference in the accuracy or variability depending on participant group.

This preliminary study on the accuracy and variability of the Weight Shift Tool will help inform further research on its validation and clinical utility for assessing weight distribution in standing for different populations.

## Methods

### Trial Design

This was a cross-sectional observational study which included convenience samples from 3 populations—healthy younger adults, healthy older adults, and people living with stroke.

### Participants

Healthy adults (18 years and older) were recruited through posters placed around the community and by word of mouth. Inclusion criteria comprised the ability to stand independently for at least 5 minutes at a time, and being free of any condition that could impair standing balance (ie, vestibular deficits, recent concussion or cervical whiplash injury, lower extremity amputation, second or third trimester of pregnancy, neurological disorders such as multiple sclerosis, severe lower extremity injury, and advanced diabetes). People living with stroke of any type (50 years and older) were recruited from the Bruyère Health inpatient stroke rehabilitation program. Inclusion criteria comprised the ability to stand independently (no gait aid or assistance, although close supervision was acceptable) for at least 5 minutes at a time, and being medically stable, without seizures or any of the previously mentioned conditions that could further impair standing balance. All participants had to have adequate vision to follow an exergame on a TV screen located 2 m away.

As this was a preliminary, observational study, no sample size calculation was performed. Sample size was determined by the availability of resources. People living with stroke were age-matched (within an average of 5 years) to healthy older adults. Because of limited resources, the sample size was not large enough to allow for subgroup analyses. The following demographic information was collected: sex, weight, height, and age, as well as type of stroke and hemiplegic side for people living with stroke.

### Equipment

The percentage of weight on each foot assessed by the Jintronix Rehabilitation System Weight Shift Tool was compared to that assessed by the MatScan pressure mat (Tekscan, Inc).

**Figure 1:**
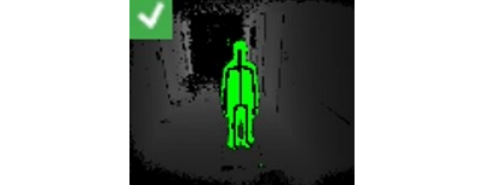
Skeleton of body as seen by the Kinect v2 sensor and Jintronix software.

The Weight Shift Tool is an assessment tool included within the Jintronix Rehabilitation System nonimmersive virtual reality platform. The Jintronix Rehabilitation System was originally designed for clinician-led stroke recovery, and includes a variety of customizable games, activities and exercises designed to address standing and sitting balance, gait, and upper extremity movement. The Jintronix Rehabilitation System is based on MMC using the Kinect v2 sensor hardware to interact with the user. The Kinect v2 sensor uses 2 cameras (both sampled at 30 Hz), a red, green, and blue visual camera and an infrared projector combined with an infrared detector that receives returning infrared light reflected from objects [27]. The more distant the object, the longer the “time of flight” between projection and detection, allowing for depth detection. The associated software, Windows Software Development Kit (Microsoft Corp), uses the cameras to detect 20 joints on a skeleton and therefore recognize gestures and motion (Figure 1). The Jintronix Rehabilitation System uses a proprietary algorithm to use the position of the joints to estimate the percent weight on each foot, which is unavailable publicly.

**Figure 2:**
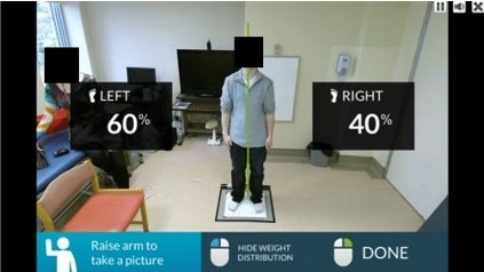
Screen capture of the Weight Shift Tool showing a participant with the percentage of weight borne on each foot visible on the screen.

The Weight Shift Tool measures the center of pressure kinematically to estimate the percentage of weight on each foot. The user stands 1.90-2.20 m from the Kinect v2 sensor within a 0.30 m by 0.30 m area and sees a mirror image of themselves on the screen in real time. The Weight Shift Tool determines the midline of the body (green line in Figure 2), and the degree that this line is off the vertical is translated into an estimation of the percentage of weight on each foot. The percentage can be shown on the screen (Figure 2) or hidden. The percentages are updated in real time as the user’s weight shifts. The user is prompted to take a point-in-time capture of the percentage of weight on each foot by raising their arm vertically. Recordings are stored in the online Jintronix database. To record the percentage of weight on each foot without raising the arm for the purposes of this study, Movavi Video Suite Personal 14 movie editing software (Movavi Software Inc) was used for single point-in-time screen capture of the Jintronix computer screen, triggered by a computer-keyboard hot key.

While testing the Weight Shift Tool, participants stood on a MatScan pressure mat, which measures pressure to estimate the percentage of weight on each foot. The MatScan pressure mat consists of a 44 by 52 array of sensels, each measuring 0.84 cm by 0.84 cm. The mat was calibrated to each participant’s weight and the pressure under the feet was recorded at one point in time, triggered by a computer keyboard hot key. MatScan Research Software (Tekscan Inc) was used to position a divider between the 2 feet and calculate the percentage of weight under each foot. Although a force plate is regarded as the gold standard for measuring the forces produced in stance, the MatScan pressure mat has been validated against the force plate for healthy, younger adults (*r*=0.92-0.99 and *P*<.05 for all center-of-pressure variables during single-leg stance with eyes-open and eyes-closed trials; *r*=0.85-0.98 and *P*<.05 for the average percent change for all center-of-pressure variables between eyes-open and eyes-closed trials) and has been deemed reliable for measuring postural stability in older adults with rheumatoid arthritis (intraclass correlation coefficient=0.84 (95% CI 0.63-0.93) to 0.92 (95% CI 0.81-0.97), SEM=1.27-2.35 mm) [28,29]. The pressure mat was chosen for this study because it is more feasible to use in a clinical setting than a force plate.

### Procedure

**Figure 3:**
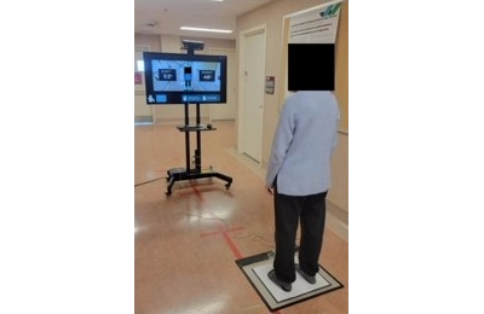
Experimental set-up with the participant standing on the pressure mat.

Demographic data were documented for all participants, and people living with stroke were administered the Berg Balance Scale to characterize their balance ability [30]. All participants wore pants and a shirt (ie, no skirts) and stood in sock feet on the pressure mat, in a comfortable stance, with hands by their side and eyes looking at the Jintronix screen (Figure 3). The position of the participant’s feet on the pressure mat was drawn on a piece of paper taped to the pressure mat during the initial stance so that the exact foot placement would be reproduced for the following stances. Two series of 9 stances were captured; the order of the series was randomized. In the first series, the Jintronix view of the percentage of weight on each foot was activated but covered on the screen, so that the participants could not see the percentages. Participants held the required position, and a screen capture and pressure mat recording were taken simultaneously. In the second series, the Jintronix view of the percentage of weight on each foot was deactivated, and the pressure mat recording was taken as the participant raised their arm with the shoulder in 90 degrees of abduction and the elbow in 90 degrees of flexion to capture the Weight Shift Tool recording (as seen on the TV screen in Figure 2). Participants could not be blinded to their arm raise condition. In both series, participants were verbally instructed to “stand with equal weight bearing on both feet,” “stand with more weight on the left foot,” or “stand with more weight on the right foot.” People living with stroke completed the tasks to their ability, even if it was not possible for them to put more weight on one foot. Each stance was repeated 3 times in each series, presented in a randomized fashion. All stances were completed by all participants. Participants could take a rest (standing or sitting) during testing, ensuring that their feet were repositioned accurately on the footprints. The purpose of measuring recordings with arm raised (AR) and with no arm raised (NAR) was to determine if the use of the Jintronix software’s recommended means of capturing a recording (ie, by raising the arm) would alter measurements of weight distribution by disturbing the participant’s natural stance or by exaggerating or diminishing the participant’s lean. Therefore, both arm raise conditions were compared.

### Analysis

The pressure mat recordings and Weight Shift Tool recordings from the left foot were analyzed, since the percentage of weight borne on the right foot would always be the recorded percentage of weight borne on the left foot subtracted from 100%, and consistent analysis of the same foot was necessary. An average of the 3 repetitions for each series (AR or NAR), lean type and participant was calculated and used for analysis. Each of the 3 participant groups (younger adults, older adults, and people living with stroke) was analyzed separately. Bland-Altman analyses were performed using Microsoft Excel (Microsoft) to assess agreement between the pressure mat recordings and each method of capturing Weight Shift Tool recordings (raising arm to record, or screen capture without raising arm) [31]. Bland-Altman analysis involves plotting the difference between paired measurements of 2 different methods of clinical assessment (*y*-axis) against the means of the paired measurements (*x*-axis). This allows for an assessment of the agreement of the 2 methods (ie, how interchangeable they may be). The mean of all the differences represents the bias (ie, the average difference between the 2 methods). If the 95% CI for the bias includes zero, then the mean percent weight on the left foot as measured from the Weight Shift Tool is statistically equivalent to the mean percent weight on the left foot as measured from the pressure mat. If the 95% CI for the bias does not include zero, then the Weight Shift Tool will not be considered accurate compared to the pressure mat. The “limits of agreement” of a Bland-Altman plot represent the 95% CI of the distribution of the differences between the 2 methods. If the bias is significantly different from zero, or the limits of agreement are greater than 5% weight on the left foot, then the 2 methods may not be used interchangeably. The acceptable range of variability is informed by a study by Martello et al [32], who established a minimal detectable change (MDC) of 5% for weight-bearing symmetry in people living with stroke. Therefore, limits of agreement exceeding 5% would mean that variability caused by the Weight Shift Tool could be misconstrued as a detectable change of weight distribution in standing. Exceeding this threshold would render the Weight Shift Tool unsuitable for measuring weight distribution in standing, as changes detected by it may not represent true changes in weight-bearing symmetry.

To investigate the impact of participant group, lean, and arm raise on the data, a 3-way mixed methods ANOVA was performed with SPSS (version 28; IBM Corp). The dependent variable was the difference between the percentage of weight measured on the left foot as tested with the Weight Shift Tool and with the pressure mat. The between-subject factor was group. Within-subject factors were the direction of lean and the presence of arm raise. Post hoc analyses were performed using univariate ANOVAs. Statistical significance was defined as *P*<.05.

Data are available from the authors upon reasonable request.

### Ethical Considerations

The study was approved by the Research Ethics Boards at Bruyère Health (M16-15-030) and the University of Ottawa (A07-15-02). All participants provided written informed consent. Participants’ privacy and confidentiality were maintained throughout the study. Participants were provided with a CAD $5 (US $3.88) coffee shop gift card to thank them for their time.

## Results

Twelve individuals in each of the 3 groups participated. Demographic data are presented in Table 1. The mixed methods ANOVAs analyzed only 11 people living with stroke, due to 1 participant leaning to the incorrect side during every right lean.

**Table 1:** Demographic data for study participants.

Participant group	Number	Sex	Age (years), mean (SD)	BMI (kg/m^2^), mean (SD)	Hemiplegic side	Type of stroke	Berg Balance Scale (out of 56), mean (SD)
Younger adults	12	7 male 5 female	22.9 (3.5)	23.7 (3.2)	N/A	N/A	N/A
Older adults	12	2 male 10 female	67.2 (9.3)	25.3 (3.7)	N/A	N/A	N/A
People living with stroke	12	10 male 2 female	69.8 (13.5)	26.2 (4.8)	6 right 6 left	8 ischemic 2 hemorrhagic 2 unknown	48.7 (8.4)

**Figure 4:**
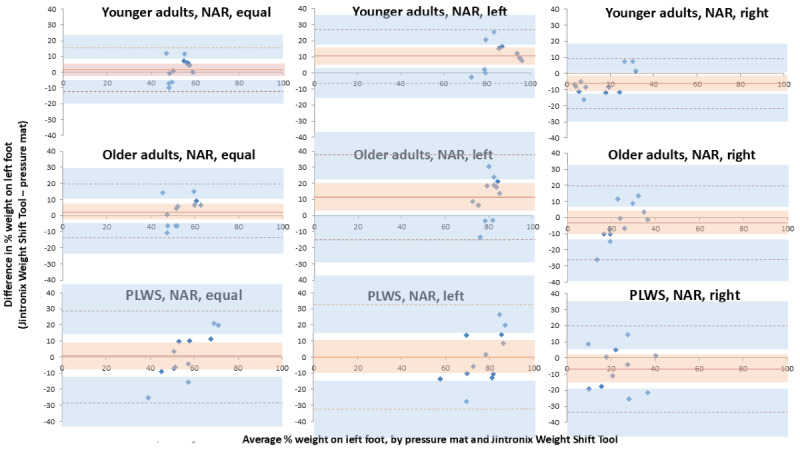
Bland-Altman plots for younger adults, older adults, and people living with stroke, showing 3 stance positions (equal stance, left lean, and right lean), without raising an arm to trigger a screenshot (no arm raise). The solid orange line represents the bias between the Weight Shift Tool and the pressure mat. Dashed orange lines represent the limits of agreement for the bias. The orange area represents the 95% CI for the bias. The blue area represents the 95% CIs for the limits of agreement. NAR: no arm raise; PLWS: people living with stroke.

**Figure 5:**
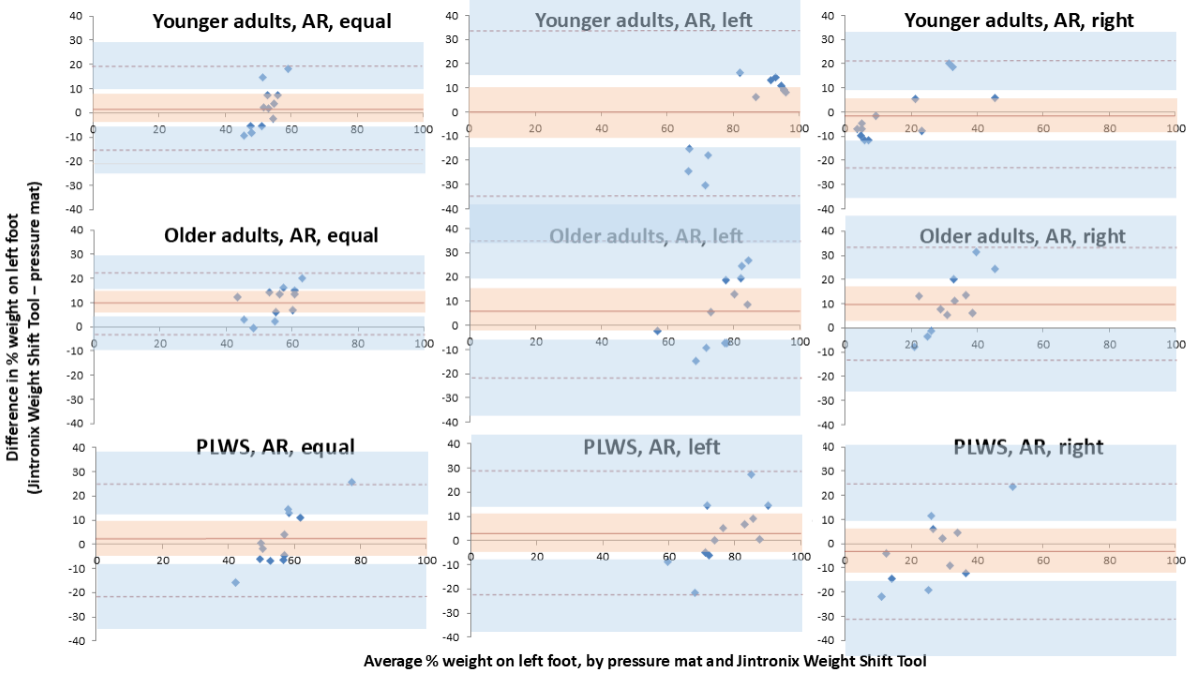
Bland-Altman plots for younger adults, older adults, and people living with stroke, showing 3 stance positions (equal stance, left lean, and right lean), raising an arm to trigger a screenshot (arm raise). The solid orange line represents the offset between the Weight Shift Tool and the pressure mat. Dashed orange lines represent the limits of agreement for the bias. The orange area represents the 95% CI for the bias. The blue area represents the 95% CIs for the limits of agreement. AR: arm raise; PLWS: people living with stroke.

Bland-Altman plots were prepared for each participant group (younger adults, older adults, and people living with stroke), separately for NAR (Figure 4) and AR (Figure 5). Overall, for all groups, leans and arm raises, the bias between the Weight Shift Tool and the pressure mat was small to moderate (0.0%-11.7% of body weight on the left foot) (Tables 2-4). However, limits of agreement were generally wide (12.8%-33.6% above and below the bias), indicating high levels of variability. With AR, younger adults had the smallest average bias of all participant groups, in equal (1.9%), left (0.0%), and right (−0.9%) stances. This was not the case with NAR, where no participant group consistently had the lowest average bias across all stances. Biases were smallest for people living with stroke equal stance NAR (0.6%), people living with stroke left lean NAR (0.2%), younger adults left lean AR (0.0%), and younger adults right lean AR (−0.9%). Across all lean conditions and both arm raise conditions, bias was greatest for older adults except for right lean NAR.

**Table 2:** Percent weight on the left foot as measured by the Weight Shift Tool and the pressure mat for younger adults. Means, SD, bias, and limits of agreement of 3 trials.

Arm raise condition and stance		Weight on left foot measured by Weight Shift Tool, mean% (SD)	Weight on left foot measured by pressure mat, mean% (SD)	Bias between Weight Shift Tool – pressure mat, % (limits of agreement)	
**No arm raise**					
	Equal weight on both feet	53.6 (6.7)	51.6 (4.4)	1.9 (−12.1 to 16.0)	
	Left lean	92.1 (9.9)	81.8 (8.4)	10.4 (−6.2 to 27.0)	
	Right lean	12.6 (13.5)	18.5 (8.8)	−5.9 (−21.1 to 9.4)	
**Arm raise**					
	Equal weight on both feet	53.2 (7.6)	51.2 (2.9)	2.0 (−15.6 to 19.5)	
	Left lean	84.3 (19.6)	84.3 (6.2)	0.0 (−33.6 to 33.6)	
	Right lean	15.8 (18.8)	16.7 (10.6)	−0.9 (−23.2 to 21.3)	

**Table 3:** Percent weight on the left foot as measured by the Weight Shift Tool and the pressure mat for older adults. Means, SD, bias, and limits of agreement of 3 trials.

Arm raise condition and stance			Weight on left foot measured by Weight Shift Tool, mean% (SD)		Weight on left foot measured by pressure mat, mean% (SD)		Bias between Weight Shift Tool – pressure mat, % (limits of agreement)
**No arm raise**							
	Equal weight on both feet	54.5 (8.8)		51.8 (5.6)		2.7 (−14.3 to 19.7)	
	Left lean	85.9 (9.0)		74.2 (5.8)		11.7 (−14.2 to 37.6)	
	Right lean	22.8 (12.2)		26.0 (5.2)		−3.2 (−26.4 to 20.0)	
**Arm raise**							
	Equal weight on both feet	60.0 (8.5)		49.8 (5.3)		10.3 (−2.6 to 23.1)	
	Left lean	79.6 (13.7)		73.3 (6.6)		6.3 (−22.4 to 34.9)	
	Right lean	36.7 (12.4)		26.8 (5.0)		9.9 (−13.1 to 33.0)	

**Table 4:** Percent weight on the left foot as measured by the Weight Shift Tool and the pressure mat for people living with stroke. Means, SD, bias, and limits of agreement of 3 trials.

Arm raise condition and stance		Weight on left foot measured by Weight Shift Tool, mean% (SD)	Weight on left foot measured by pressure mat, mean% (SD)	Bias between Weight Shift Tool – pressure mat, % (limits of agreement)
**No arm raise**				
	Equal weight on both feet	56.3 (16.0)	55.7 (5.6)	0.6 (−27.9 to 29.1)
	Left lean	77.1 (15.4)	76.8 (7.9)	0.2 (−32.2 to 32.7)
	Right lean	20.2 (11.7)	26.5 (12.3)	−6.3 (−33.2 to 20.6)
**Arm raise**				
	Equal weight on both feet	57.1 (14.0)	54.8 (4.7)	2.2 (−21.2 to 25.6)
	Left lean	78.5 (14.3)	75.5 (6.9)	3.0 (−22.7 to 28.7)
	Right lean	25.7 (17.1)	28.6 (9.0)	−2.9 (−30.9 to 25.1)

The acceptable threshold for limits of agreement (±5%) was exceeded for all conditions. Across groups, variability was greatest for people living with stroke in all lean and arm raise conditions except for left lean AR, with limits of agreement ranging from 23.4% to 32.5% above and below the bias. On average, limits of agreement were narrower with equal weight on both feet (ranging from 23.4% to 28.5% above and below the bias) than with right lean (ranging from 26.9% to 28.0% above and below the bias) or left lean (ranging from 25.7% to 32.5% above and below the bias). With equal stance, bias was greatest for older adults, for both AR (10.3%) and NAR (2.7%). With equal stance, NAR produced a smaller bias (ranging from 0.6% to 2.7%) than AR (ranging from 2.0% to 10.3%).

There was no 3-way interaction between group, lean and arm raise (*P*=.12), and no 2-way interaction between group and lean (*P*=.82). There was, however, a 2-way interaction between group and arm raise (*P*=.02) and between lean and arm raise (*P*=.004). While there was no effect of group when there was no arm raise (*P*=.45), there was an effect when the arm was raised (*P*=.04), such that there was less bias in younger adults compared to older adults (Figures 4 and 5). Lean direction had an effect on the bias when the arm was not raised (*P*<.001), such that there was a smaller bias with equal weight-bearing, a tendency to overestimate when leaning to the left, and a tendency to underestimate when leaning to the right (Figure 4). Combining all participant groups and with the arm not raised (Figure 4), the bias during equal weight-bearing versus left lean was similar (*P*=.10). However, equal weight-bearing versus right lean was significantly different (*P*=.04), as were left versus right lean (*P*<.005). There was no effect on the bias from lean directions when the arm was raised (*P*=.67; Figure 5).

## Discussion

### Principal Findings

None of the study’s hypotheses were confirmed by the results. This study found that, on average, for all stances and groups, the bias between the Weight Shift Tool and the pressure mat was small to moderate and proved not to be statistically significant. However, Bland-Altman analysis showed that variability was high across all groups. In older adults and people living with stroke, the limits of agreement of all stances exceeded acceptable levels of variability (limits of agreement≤±5%). In younger adults, the limits of agreement of all stances were generally narrower and the average bias was the smallest of each participant group for all stances, although still beyond acceptable levels of variability. This suggests that the Weight Shift Tool’s proprietary algorithm for estimating weight shift may have been trained using a population of healthy, younger adults and adjusted for accuracy when raising the arm, as originally intended for its use. Variations in posture, body shape, and weight distribution strategies may explain the greater biases of older adults and wider limits of agreement for people living with stroke, as these factors may affect the ability of the Weight Shift Tool to accurately estimate the percentage of weight borne on each foot due to its method of estimating asymmetry. For example, by kinematically monitoring deviation from the vertical of central body segments, the Weight Shift Tool may not interpret a change in weight distribution if a person increases the weight under one foot by bending the ipsilateral knee. Furthermore, for all groups, the limits of agreement were narrower when standing with equal weight on both feet. This shows that the Weight Shift Tool is more variable with drastic asymmetries in weight-bearing, also noted as there were instances in which the Weight Shift Tool estimated that 100% of a participant’s weight was on one foot when leaning, although this was not the case. With no arm raise, a statistically significant difference was noted for right lean (tendency to underestimate bias) versus left lean (tendency to overestimate bias), as well as right lean versus equal stance (smaller bias), but not for left lean versus equal stance. This trend was unexpected and could be due to the algorithm of the Weight Shift Tool compensating for differences in symmetry when raising the arm, as this is the designed method for capturing recordings.

Previous studies have compared the Kinect sensor or other MMC to a pressure mat or force plate. Dolatabadi et al [33] validated the Microsoft Kinect sensor for Windows v2 against the GAITRite pressure mat for the analysis of gait parameters (velocity, stance time, step length, and step time) in healthy young adults. Gonzalez et al [34] found that fewer stabilometric parameters of the center of mass measured by the Kinect v2 sensor detected body sway compared to parameters of center of pressure and center of mass measured by a force plate. Dehbandi et al [35] found that the centers of pressure measured by the Kinect v2 sensor and a force plate during a seated balance task in healthy participants were strongly correlated. As the Kinect v2 sensor was deemed sufficiently accurate, they created an analytical algorithm based on machine learning for the assessment of postural stability. The Vicon MMC system was deemed accurate for estimating ground reaction forces in comparison to a force plate (average root-mean-square difference of 0.75 N/kg across all dimensions and tasks, *R*^2^=0.93-0.99 for peak forces across tasks) [36]. Bland-Altman analysis of the Qualisys MMC system compared to a force plate for evaluating the margin of stability during gait initiation generally showed broad limits of agreement (>20%), which were lower in healthy young adults, greater in healthy older adults, and greatest in patients with Parkinson disease [37]. Therefore, not all MMC systems appear to demonstrate comparable accuracy and variability. Although there is precedent work evaluating the accuracy and variability of the Kinect sensor and other MMC, this is the first study to compare the percentage of weight on each foot as measured by the Kinect sensor and the pressure mat.

The findings of this study are in line with previous studies on the accuracy of the Kinect sensor in detecting movements in the mediolateral direction. Several authors found that the Kinect v2 sensor measured trunk lean and body sway less accurately in the mediolateral direction than in the anteroposterior direction compared to the Vicon marker–based motion tracking system, particularly during double-leg tests [38,39]. Clark [40] found that lateral trunk lean angles measured with the Kinect sensor were inaccurate when tested against the Vicon marker–based motion tracking system without calibration (mean error of 3.2°, SD 2.2°) [40]. Individualized calibration (which was also done in this study) significantly improved the comparison (mean error of 0.8°, SD 0.8°). Thus, the conclusion that the Weight Shift Tool was not accurate in the mediolateral direction is confirmed by other studies evaluating the Kinect sensor. Furthermore, Luizzo et al (2017) [41] evaluated the reliability of the Wii Fit balance board for measuring weight-bearing asymmetry in older adults and people living with stroke. The study determined that the MDC for older adults and people living with stroke was 8.8% and 13.6%, respectively, which were both larger than the MDC established by Martello et al [32]. The authors concluded that the MDC was sufficiently large as to deem the Wii Fit balance board unreliable for measuring weight-bearing asymmetry in older adults and people living with stroke with factory settings [41]. This supports the study’s appraisal of the Weight Shift Tool as inaccurate for older adults and people living with stroke, due to limits of agreement exceeding the established MDC of 5%. Clinicians require tools that provide accurate and consistent measurements to inform treatment choices and progressions, and to predict outcomes. Limits of agreement greater than an established MDC, and inconsistency in the bias (especially with older adults) indicate that the Weight Shift Tool is too variable for clinical use, as a patient may not, for example, show a real improvement where one exists. An MMC should not have limits of agreement greater than 5%, and the Weight Shift Tool is far from this threshold [32,41].

### Limitations

A limitation of this study is the small sample size, which would make the 3-way ANOVA less likely to determine differences and may increase the limits of agreement in the Bland-Altman analysis. As well, it was not possible to perform subgroup analyses, limiting the generalizability of the results. In addition, the study sample is not representative of the entire stroke population due to the nature of the task. However, individuals who did not meet the inclusion criteria (ie, the ability to stand without assistance or a gait aid) would not use this tool clinically. Therefore, the study sample represents only those for whom the Weight Shift Tool is suitable.

Another limitation is that this study did not take into account which arm was being raised by participants, the dominant leg, or the side of stroke for people living with stroke. The arm that a participant raised during the AR trials could have had an impact on the measurement of weight distribution on each foot. Nevertheless, the arm was not raised overhead, but only to 90 degrees of shoulder abduction with the elbow at approximately 90 degrees, as demonstrated by the Weight Shift Tool during use (Figure 2).

Furthermore, it would have been ideal for leans of varying extents to be tested, rather than solely instructing the participants to “lean to the left (or right).” Additionally, different body shapes, weight distribution strategies, and postures could have been compared to determine if variations would affect the estimation of lean. Finally, there is no minimal clinically important difference data available for comparison.

### Future Directions

This preliminary study has identified recommendations for the improvement of the Jintronix Weight Shift Tool, as well as direction for more in-depth evaluations of the accuracy and variability of MMC systems for the measurement of weight distribution in standing.

With respect to the Jintronix Weight Shift Tool, developments in the proprietary software, which analyzes the figure to determine weight distribution, and hardware, which captures the figure for software analysis, could offer improvements. In order to be acceptable for clinical use, the removal of statistical bias and reduction of the limits of agreement to 5% would be required. Recommended improvements include algorithmic calibration adjustments and additional model training (possibly including force plate ground truth for improved computational modeling) to increase accuracy for the estimation of lean from the vertical for varying body shapes, postures, and distribution strategies. Tracking additional landmarks in the trunk and including a measurement of height and BMI for calibration may help as well. Additionally, given that the Weight Shift Tool is intended to be used in the presence of a clinician, removing the need for raising the arm may improve the accuracy of estimates.

With respect to future studies, repeated measures may provide a more accurate average weight distribution, particularly for younger adults and people living with stroke when their arm is used to trigger a recording, as well as for people living with stroke when their arm is not raised, since the bias was small in these instances. Moreover, subgroup analyses could be performed to determine if individual differences, such as BMI and sex, affect results. A trial design establishing the MDC or minimal clinically important difference for weight-bearing on each foot would allow for a more thorough evaluation of the clinical acceptability of the Weight Shift Tool. Future studies on the ability of the Weight Shift Tool to assess change over time could also better inform the use of the Weight Shift Tool as an outcome measure for research.

While the Weight Shift Tool may be able to track changes in weight distribution in real time (ie, in the context of gaming), its accuracy is poor and it cannot be recommended for clinical use. Currently, other non-MMC tools for measuring weight-bearing asymmetry, such as smart insoles, may also help understand the usefulness of the Weight Shift Tool in comparison to other tools [42].

### Conclusions

The objectives of this study were to assess the accuracy and variability of the Weight Shift Tool in comparison to a pressure mat, and to determine if the participant group or raising the arm would affect the accuracy or variability. To do so, the percentage of weight on each foot was measured by both tools in younger adults, older adults, and people living with stroke, when leaning to the left, leaning to the right, or with equal weight-bearing, and with an arm raised or no arm raised. The findings suggest that the bias of the Weight Shift Tool compared to the pressure mat may be accurate for healthy younger adults with an arm raised but the Weight Shift Tool is much too variable for clinical use for all tested groups. Unacceptable levels of bias for older adults and high variability for people living with stroke resulted in poor agreement between the Weight Shift Tool and the validated MatScan pressure mat for these populations. Therefore, these preliminary findings do not support the recommendation of the Weight Shift Tool for clinical use for measuring weight distribution in standing in younger adults, older adults, or people living with stroke. The findings of this study are specific to the Jintronix Rehabilitation System Weight Shift Tool with the Microsoft Kinect v2 sensor. Future versions of this MMC may have enhanced capabilities to measure weight distribution among various populations. Therefore, further research would be required to validate MMC systems for measuring weight shift as a clinical assessment and rehabilitation tool.

## Abbreviations

AR: arm raise
MDC: minimal detectable change
MMC: markerless motion capture
NAR: no arm raise
